# Supplementary material for: Killing a zombie: a full deletion of the BUB1 gene in HAP1 cells
Source: EMBO J. 2019 Oct 22;38(22):e102423. doi: 10.15252/embj.2019102423 (PMC6856604; doi:10.15252/embj.2019102423)
Supplement: Supplementary file 1 — Appendix [file EMBJ-38-e102423-s001.pdf]

Table of Contents

Appendix Material and Methods
Appendix Table S1
Appendix References

Appendix Materials and Methods

Cell Culture, siRNA and Drug Treatments

HAP1 cells were cultured in Iscove's Modified Dulbecco's Media (IMDM) (Gibco) supplemented with 10% fetal calf serum (FCS), 100 U/mL penicillin, and 100 mg/mL streptomycin. Nocodazole, Noscapine and Reversine were dissolved in DMSO and were used at the indicated concentrations. For siRNA, HAP1 cells were transfected using Lipofectamine RNAiMAX (Thermo Fisher) according to manufacturer's guidelines. Cells were either mock transfected or transfected with 20nM of BUB1 siRNA (5'-GAGUGAUCACGAUUUCUAA -3', Dharmacon) 48 hours prior to the start of the live cell imaging experiments. After 18 hours, cells were washed twice with culture medium.

Generation of full BUB1 deletion clones

To generate a full BUB1 gene deletion in HAP1 cells, guide RNA's were designed against exon 1 and exon 24 of the BUB1 gene (see Table S1 for gRNA sequences). gRNA's were designed using CRISPOR (Haeussler *et al*, 2016). Guides were cloned into the pX330 vector (Cong *et al*, 2013). Cells were reverse transfected using FuGene HD (Promega) according to the manufacturer's protocol. A vector containing a puromycin resistance-cassette was co-transfected and cells were selected with 2μg/mL puromycin 2 days-post transfection. Individual clones were picked and validated by genomic PCR, RT-PCR and western blot analysis.

Genomic PCR

Hap1 cells were grown in 6 cm culture plates and cells were harvested by trypsinization. Cell pellets were lysed in 50μL of DirectPCR lysis reagent (Viagen) supplemented with proteinase K. Lysates were incubated at 55 °C for 4 hours and proteinase K was inactivated by incubating the lysates at 85 °C for 45 minutes. Product were amplified using MyTaq DNA polymerase. Primers are listed in Table S1.

RT-PCR

Whole-cell RNA was purified from HAP1 using the Qiagen RNA Easy kit according to the manufacturer's guidelines. cDNA was synthesized with Oligo(dT)18 primers (Thermo Fisher) using BioScript (Bioline). 2 μg of RNA was used as input. RT-PCR was performed on 100 ng of cDNA per reaction and products were amplified in 30 cycles using MyTaq (Bioline). Real-time PCR primer pairs were designed with a T_m of close to 60°C to generate 180–500 bp amplicons, all spanning introns. BUB1 Ensemble transcript 202 was used as a basis to design the primers. A product that was higher than expected when amplifying exon 20-22 was observed. This indicated that HAP1 most likely express Ensembl transcript 201. Primer sequences can be found in Table S1.

Western blot

HAP1 cells were harvested and lysed using Laemmli buffer [120 mM Tris (pH 6.8), 4% SDS, 20% glycerol]. 25 μg of protein was separated on a polyacrylamide gel and subsequently transferred to nitrocellulose membranes using semi-dry transferring (Trans-Blot, Biorad). Membranes were blocked for 1 hour at RT in 5% milk/PBS. Primary antibodies were incubated in 2,5%milk/PBST overnight at 4°C and peroxidase-coupled secondary antibodies (DAKO, 1:2000) were incubated for 2 hours at room temperature. Antibodies were visualized using enhanced chemiluminescence (ECL) (Immobilon) in a ChemiDoc Imaging system (Biorad). The following antibodies were used: mouse anti-α-tubulin (Sigma, t5168), mouse anti-BUB1 (Abcam, ab54893), rabbit anti-pH2A-Thr120 (Active Motif, 39391).

Immunofluorescence

Cells were grown on 10 mm glass coverslips and pre-extracted for 60 s in PEM buffer (100 mM PIPES, 10 mM EGTA, 1 mM MgCl, and 0.1% Triton X-100), followed by fixation for 10 min at room temperature in 4% formaldehyde in PEM buffer with 0.3% Triton X-100. All primary antibodies were incubated overnight at 4°C. The following antibodies were used: human anti-ACA (Cortex Biochem), rabbit anti-pH2A-Thr120 (Active Motif, 39391), mouse anti-BUB1 (Abcam, ab54893), rabbit anti-BUBR1 (Bethyl, A300-386A). Secondary antibodies (1:600, Molecular Probes, Invitrogen) and DAPI were incubated for 2 hr at room temperature. Coverslips were mounted using Vectashield (Vector Laboratories). Images were acquired using a Deltavision deconvolution microscope (Applied Precision) with a 60x 1.42 numerical aperture (NA) oil objective. Softworx (Applied Precision), ImageJ, Adobe Photoshop, and Illustrator CS6 were used to process acquired images.

Time-Lapse Microscopy

Cells were plated on 8-well glass-bottom dishes (LabTek). 3 hours prior to imaging, cells were incubated with 250nM SiR-DNA (Spirochrome) to stain the DNA. Cells were imaged using a CO2-controlled Deltavision deconvolution microscope (GE Healthcare) equipped with a heat chamber. Images were acquired every 5 min using a 40x (1.3 NA) objective. Z-stacks were acquired with 3-mm intervals. Images were analyzed and processed using Softworx and ImageJ.

Appendix Table S1. gRNA sequences and primer sequences

Guide RNA's	Forward	Reverse
BUB1 Exon 1	CACCGTCCTTCAGTAAGTGTCCGTC	AAACGACGGACACTTACTGAAGGAC
BUB1 Exon 24	CACCGAGATTAGGGCCCTACGTAAT	AAACATTACGTAGGGCCCTAATCTC

genomic PCR	Forward	Reverse
MAD2	GTGCTGCGTCGTTACTTTTG	GGGACGGATCTGCACTTAAA
BUB1 Exon 1	GGCTTCTAGTTTGCGGTTCA	ACTGTCAGGAGGGGATTCCT
BUB1 Exon 10	AGCTTATTCGTGGGGAATCA	GCAAACGCAAAATGGGTTA
BUB1 Exon 15	TCAGGCTCCTACACTTCCTGA	CACACACATTGAGTACCTTGTCA
BUB1 Exon 23	GACATGTTAGTGGGGCGATT	CCAGATGGAACCCAACATTC
BUB1 Exon 24	GGAGCACAATTCCTTTGCAT	TCTCACAAATGCTTGCATCC

RT-PCR	Forward	Reverse
BUB1 Exon 2-3	AAGCCCACATGCAGAGCTAC	TGAATCTTGGGTCATTGTGG
BUB1 Exon 4-6	GGAATTCAAAACCAGGCTGA	ATGCAGGCCATGTTATTTCC
BUB1 Exon 6/7-9	CAGGGTTCAGAGCTTTCTGG	GGATGTCTCCACCACCTGAT
BUB1 Exon 10-12	TCCTGTTGTTCCTCCTTTGG	TTCCCAGTGATGTGTTTGGA
BUB1 Exon 13-16	GATGCATTTGAAGCCCAGTT	GGAAGCTTGTGGAATGGTGT
BUB1 Exon 17-19	CAGTGTACCCAGGCGACTTT	TGGGTTCCCAACAATGAAGT
BUB1 Exon 20-22	TACGAAGCTACCCAGGGAGA	TTCCATGGTTTGTTGCTGAG
BUB1 Exon 23-24	CGATTACTTTGGGGTTGCTG	GCTTTTGCCTTAACAAATCCA
B-actin	AGAAAATCTGGCACCACACC	AGAGGCGTACAGGGATAGCA

Appendix References

Cong L, Ran FA, Cox D, Lin S, Barretto R, Habib N, Hsu PD, Wu X, Jiang W, Marraffini LA & Zhang F (2013) Multiplex genome engineering using CRISPR/Cas systems. *Science* **339:** 819–823

Haeussler M, Schönig K, Eckert H, Eschstruth A, Mianné J, Renaud J-B, Schneider-Maunoury S, Shkumatava A, Teboul L, Kent J, Joly J-S & Concordet J-P (2016) Evaluation of off-target and on-target scoring algorithms and integration into the guide RNA selection tool CRISPOR. *Genome Biology*: 1–12
